# Injuries in humans caused by mantis shrimp or siriboia (Crustacea: Stomatopoda)

**DOI:** 10.1590/0037-8682-0858-2020

**Published:** 2021-04-28

**Authors:** Antonio Lucas Sforcin Amaral, Antonio Leão Castilho, Vidal Haddad

**Affiliations:** 1 Universidade Estadual Paulista, Instituto de Biociências de Botucatu, Departamento de Zoologia, Botucatu, SP, Brasil.; 2 Universidade Estadual Paulista, Faculdade de Medicina de Botucatu, Departamento de Dermatologia e Radioterapia, Botucatu, SP, Brasil.

**Keywords:** Arthropoda, Crustacea, Injuries, First aid, Occupational diseases

## Abstract

**INTRODUCTION:**

Mantis shrimps or siriboias are crustaceans belonging to the order Stomatopoda. They are known for their strong claws, which they use for defense and capturing their prey. They are classified into two groups: the spearers, which pierce the prey with sharp projections, and the smashers, which strike their prey with high-powered punches. These animals are highly feared by fishermen, and there are frequent anecdotal reports of human injuries caused by these crustaceans.

**METHODS:**

A questionnaire about injuries in humans caused by these stomatopods was administered to 23 fishermen of Colony Z10 in Ubatuba, São Paulo state, Brazil, and a survey of the literature on injuries in humans caused by these animals was carried out.

**RESULTS:**

The fishermen consider the mantis shrimp dangerous and avoid direct contact with them on account of the associated risk. We describe five reports of human injuries caused by these animals: four by the claws and one by the tail spikes.

**CONCLUSIONS:**

We describe the first aid treatment, prevention, and recommendations for such cases and propose the distribution of educational leaflets among the fishermen colonies.

## INTRODUCTION

**FIGURE 1:**
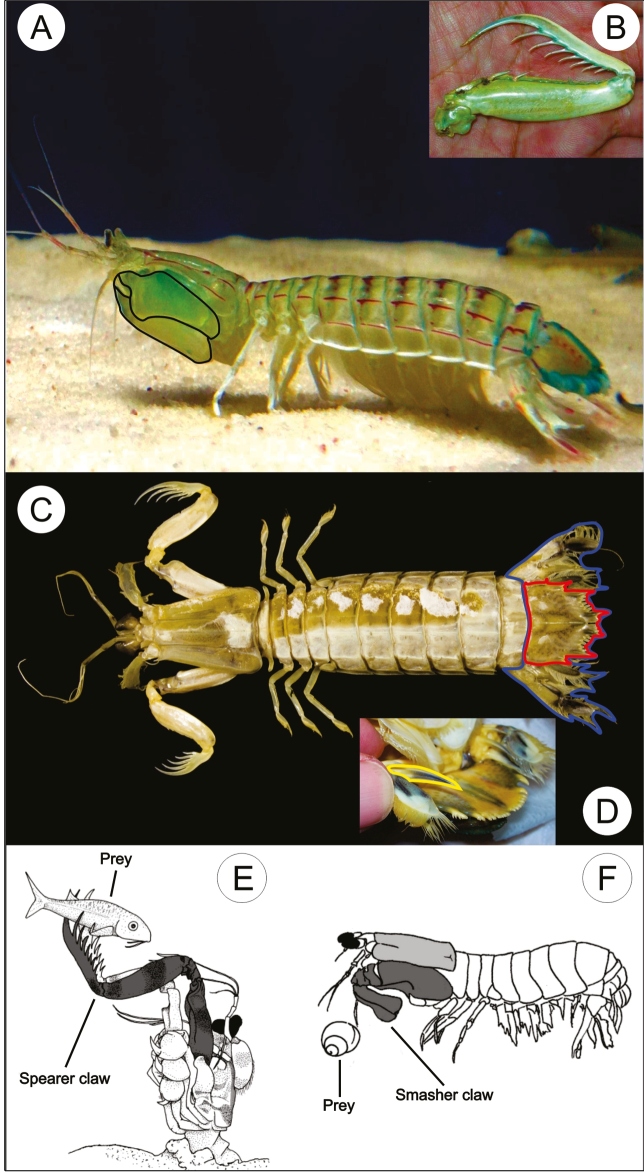
**(A)** Mantis shrimp in side view showing the raptorial claw (black outline); **(B)** The raptorial claw; **(C)** *Gibbesia neglecta* in dorsal view, showing the telson (red outline) and caudal fan (blue outline); **(D)** Sharp projection (yellow outline) on the uropod, below the telson; **(E)** A spearer stomatopod holding its prey within the sharp projections of its claw (adapted from deVries *et al.*, 2012); (F) A smasher stomatopod striking its prey (adapted from Cox *et al.*, 2014). Illustration composition (E and F adapted from Anderson *et al.*, 2014).

The mantis shrimp (Figure 1) is a marine crustacean belonging to the order Stomatopoda Latreille, 1817, and popularly known as siriboia, tamarutaca, tamburutaca, boxing shrimp, or squilla. “Siriboia” is an indigenous word formed by joining the words “*si’ri*” (crab) and *“mboi”* (snake/serpent)^1^. They are known as “mantis shrimp” because they possess raptorial claws with which they attack their prey like a mantis (Insecta: Mantodea), which strikes using its forelegs. Mantis shrimps have a global distribution and occur in tropical and subtropical waters^2^ ^,^ ^3^ ^,^ ^4^. In Brazil, there are 43 species belonging to 10 families, with a wide geographic distribution along the coast, from Amapá state (latitude 03º north) to Rio Grande do Sul state (latitude 30º south)^5^ ^,^ ^6^ ^,^ ^7^ ^,^ ^8^.

These animals stand out for their ability to strike prey with the second thoracic legs that are modified into raptorial claws (Figure 1A and B)^9^. There are two morphological types of claws that have different functions and based on which mantis shrimps are separated into two groups. The first group, known as spearers, possess claws that have three to eleven spear-like pointed projections, which are launched open when approaching the prey and close when reaching it. Thus, they capture and spear the prey with the claw-projections that pierce the body of the prey, preventing its escape (Figure 1B, C, and E)^5^ ^,^ ^9^ ^,^ ^10^ ^,^ ^11^. These animals can strike at speeds reaching 6 m/s (approximately 21 km/h)^12^. They usually occupy burrows in sandy sediments^13^ ^,^ ^14^.

The second group, known as smashers (Figure 1F), is represented by species whose claws have a big, calcified protrusion at the base, with which they strike the target at speeds reaching 30.6 m/s (about 108 km/h) with an impact of 1500 Newtons (about 152 kg), which is the acceleration equivalent to that of a projectile shot from a 9mm-caliber pistol^9^ ^,^ ^15^ ^,^ ^16^ ^,^ ^17^. These animals inhabit burrows in rocks and corals^12^ ^,^ ^13^ ^,^ ^14^ ^,^ ^18^. 

In addition to the claws, these stomatopods have two uropods, which are structures in the tail that constitute the “caudal fan” (Figure 1C). The uropods have a pair of pointed spikes each (Figure 1D) that can also be used as a weapon^14^.

The fishermen fear the “siriboia,” both in their fishnets and while walking in shallow waters during the low tide. In some parts of the Caribbean, the stomatopods are called “thumb splitter shrimp”, which highlights their ability to cause injuries^19^ ^,^ ^20^. These animals are difficult to observe. Further, the information on the injuries they cause to fishermen is limited^21^. Therefore, we aimed to identify and describe:

1. Injuries previously recorded in the literature or from unpublished data gathered by specialists on injuries caused by marine animals.
2. The occurrence of injuries in the fishermen colony, Z10 located in Ubatuba, São Paulo, Brazil, through an interview process.

## METHODS

### Bibliographic research

A literature search was performed to look for reports of injuries in humans caused by mantis shrimps, recorded by health and/or zoological professionals, with experience in fishermen colonies. The reports were studied to identify the circumstances of the injury and the aspect of the wound to understand how the injuries occur and which sequelae they can cause.

It included articles and notes published in peer-reviewed periodicals that provided information about the stomatopods and their relation with fishermen as well as books related to the subject written by researchers with experience in injuries caused by marine animals. 

### Data collection

The municipality of Ubatuba (23°26′2″ South, 45°5′9″ West) is located in the southeast region of Brazil, on the north coast of São Paulo state, in an area with open sea areas and estuaries^22^. The fishermen colony, Z10 in Ubatuba was chosen for the administration of a stratified questionnaire because it was easier to approach the fishermen here. The workers present in the colony at the time of interview were requested to answer the questions after providing consent and on confirming that they worked as fishermen in the colony. Those who did not work as fishermen in the colony were excluded. The fishermen who met the same criteria as indicated by the interviewees were also interviewed.

The questionnaire that was administered by the interviewer contained queries regarding the injuries caused by the stomatopods, together with images of the animals for proper identification. The questions asked were as follows:

- Have you heard of siriboias/tamburutacas?
- Are siriboias/tamburutacas dangerous?
- Did you suffer from injuries due to siriboias/tamburutacas?
- If yes, how and where did it happen?
- Do you have any sequelae due to the injury?
- Do you know anyone who has had an injury caused by siriboias/tamburutacas?

The answers obtained were recorded through handwritten notes and counted at the end of the interviews. Information that was not directly related to the applied question was recorded when it provided relevant facts about the interaction between the stomatopods and humans. The descriptions of four cases of injury caused by the stomatopods observed by the authors on other occasions were added to the results; they complement the validity of the fishermen’s answers obtained from the questionnaire.

### Ethics statement

The authors were granted approval to conduct studies with fishermen from the Fish Market of Ubatuba and village of Picinguaba by the Ethics Committee in Human Experimentation of Faculdade de Medicina de Botucatu, São Paulo State University, under registration CAAI 59887316.5.0000.5411, report number 1.759.505 and by the Healthcare Department of Ubatuba, São Paulo.

## RESULTS

All the 23 fishermen who were interviewed reported knowing the stomatopods. Further, all the interviewees stated that they considered these animals dangerous, although none of them had been injured or experienced sequelae. However, five fishermen (21.7%) reported knowing people who had been injured by these crustaceans. One of these cases included that of a fisherman, about 50 years old, who caught a stomatopod from a sandy substrate (Figure 1E) using a fishing rod. When trying to remove it from the hook, he held the animal by the claws to avoid being injured. However, he was struck on his hand with its tail, that caused perforations through the tail spikes, resulting in bleeding and pain. One of the interviewees reported that it is possible to manipulate the smaller specimens, when necessary, by taking hold of their abdomen and folding it, so that the claws and uropod are in contact, thereby rendering them unable to injure the hands. 

Four records of injuries caused by the stomatopods have been described and observed in other situations^20^ ^,^ ^23^; they justify the fact that it is important for fishermen to be wary of these animals.

Case 1: A 24-year-old fisherman was injured while manipulating a fishing net in the “curral” (wooden structures that trap the fish, making them easy to catch) in Salinópolis, Pará state, Brazil. The stomatopod, which he did not notice in the net, struck his hand with its claws, hitting the fifth left finger and the second right finger, causing tissue laceration and severe pain, which lasted for about an hour. He reported that he washed the area intensively with soap and water, and complete healing occurred in about one week. 

**FIGURE 2:**
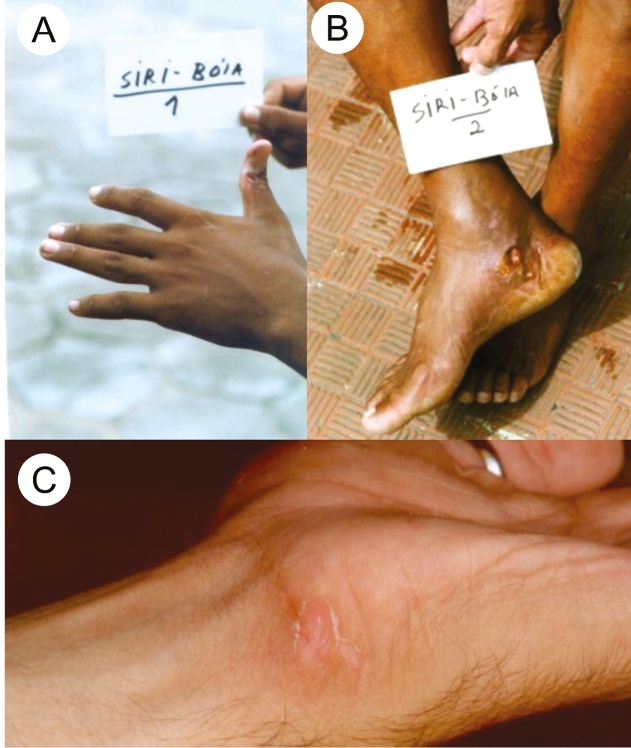
**(A)** Scars from injuries caused by a stomatopod, as described in case 2; **(B)** Injury on the right foot of a fisherman caused by a stomatopod, as described in case 3; **(C)** Injury on the left hand caused by a stomatopod, as described in case 4; tissue loss is seen in the wound due to the injury. Photo B was taken by Dr. João L. C. Cardoso.

Case 2: A 54-year-old fisherman in Ubatuba, São Paulo state, Brazil, was wounded three times on his hands by the stomatopods while fishing for shrimp. He informed that the animal was easily found in the fishing nets and had scars on his fingers that had been caused by the previous injuries (Figure 2A). He further reported that he did not undergo any treatment for the wounds when they occurred, and they persisted for weeks without healing^23^.

Case 3: A 22-year-old fisherman in Ubatuba, São Paulo state, Brazil, was wounded by a stomatopod when disembarking and removing the net with fish. He stepped on something and felt intense pain in the medial malleolus of his right foot. He reported seeing the stomatopod because the water was clear in the spot, although he was unable to capture it. The area bled heavily, and the wound persisted for months (Figure 2B). At the time of recording the interview, there was an extensive scar in the area^20^.

Case 4: A 25-year-old biology student in Ubatuba, São Paulo state, Brazil, was injured on his left hand by a stomatopod while handling a specimen during a field study (Figure 2C). The claws caused a deep laceration with heavy bleeding, resulting in an ulcer that healed after approximately three weeks. The animal was not collected for subsequent identification.

## DISCUSSION

The interviews we conducted indicate that fishermen know how these crustaceans cause injuries. They stated that the claw is dangerous, and caution is necessary to avoid getting hurt. The results showed that the stomatopods can cause injuries to humans when manipulated in fishing nets or rods, when stepped on in the sandy bottom, or in an attempt to capture them manually. The structure of the claws of both the spearers and smashers are highly specialized for capturing prey and have potential to cause injury in humans.

These animals have great strength and speed, as indicated by the injury depicted in Figure 2C; there is local tissue loss; however, it is not possible to precisely determine whether the wound was caused by a spearer or smasher. In Figure 2B, the wound appears to be deeper and has a rounded shape, which indicates a great focal impact; therefore, it might have been caused by a specimen with smasher claws. 

**FIGURE 3:**
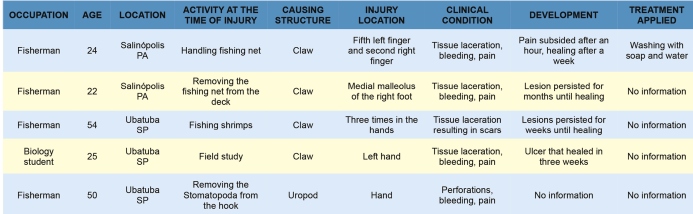
Chart with description of the cases of injuries caused by stomatopods and their outcomes.

The information that we obtained was organized in a chart to help health professionals and researchers study the data about the injuries (Figure 3).

There are no guidelines on prevention of injuries caused by the stomatopods, possibly because such incidents rarely occur. Based on the information obtained in this research, we recommend the following procedure for treating injuries caused by the stomatopods:

### Intensive washing and surgical exploration

#### Prevention of tetanus

Antibiotic therapy: Cephalexin 2 mg/ day for 10 days or amoxicillin and potassium clavulanate 1.5 mg/day for 10 days^21^.

**FIGURE 4:**
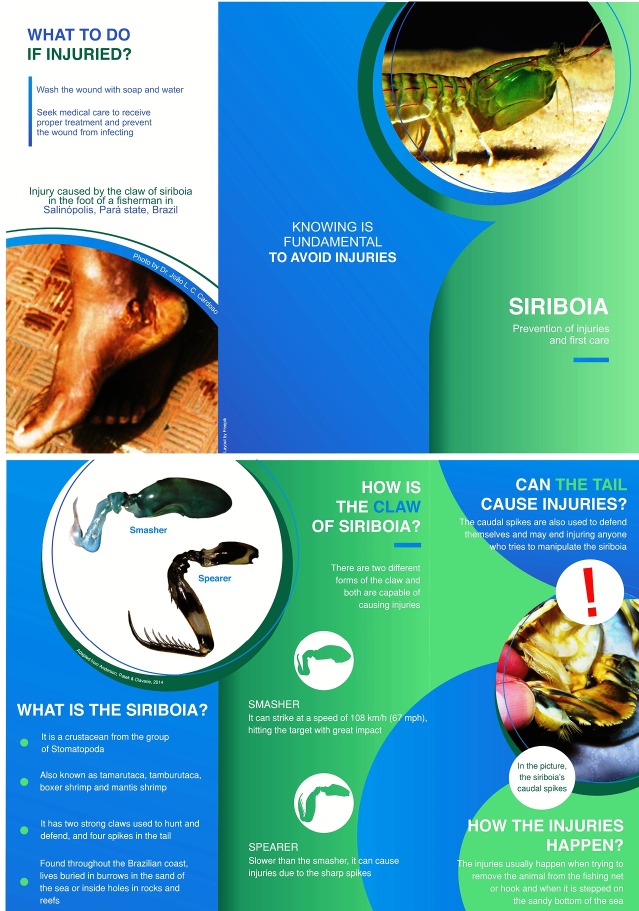
Educational leaflet about injuries caused by stomatopods.

For educational and injury-prevention purposes, we used the data obtained to prepare an informative leaflet (Figure 4) to be distributed to the fishing colony where the study was conducted. It is a compilation of information on the stomatopods, the risks associated with improper handling, and treatment protocols in case of injuries. This initiative aims to reduce the lack of information and injuries caused by the stomatopods.
